# Factors affecting the retention of healthcare assistants in English mental health services: a qualitative interview study

**DOI:** 10.1186/s12913-025-12665-1

**Published:** 2025-04-05

**Authors:** Michaela Senek, Jaqui Long, Sally Ohlsen, Rachael Finn, Scott Weich, Tony Ryan, Emily Wood

**Affiliations:** 1 School of Medicine and Population Health, The University of Sheffield, Sheffield, UK; 2 Management School, The University of Sheffield, Sheffield, UK; 3 School of Allied Health Professionals, Nursing and Midwifery, The University of Sheffield, Sheffield, UK

**Keywords:** Retention, Workforce, Healthcare assistants, Support workers, Recovery workers, Mental health services

## Abstract

**Background:**

In the UK, healthcare assistants (HCAs) work alongside registered nurses and other clinicians to provide frontline clinical care. HCAs provide a considerable amount of essential direct patient care which, dependent on the setting, can include monitoring of temperature, pulse, respirations and ECGs, support with daily activities, emotional support and facilitating communication with other health professionals. In 2019, the leaver rate of HCAs and support workers in the UK was 13.4%. In many Trusts this was higher than the leaving rate for nurses. The aim of this study was to explore HCAs’ experiences and their perceptions of the reasons for poor retention rates.

**Methods:**

We recruited HCAs from three English mental health Trusts. Recruitment information was circulated using a variety of approaches. 31 participants took part in semi-structured interviews. We explored the factors they considered to affect HCAs’ decision to leave their role, and any differences they perceived with registered staff. Interviews were coded and analysed using the framework generated in a previous phase of the study which focused on retention of registered mental health professionals.

**Findings:**

Three key factors impacted HCAs job satisfaction, wellbeing, and motivation to remain in post: (1) high workloads and unclear role boundaries creating stress and concerns for patient care; (2) good relationships with line managers and colleagues providing essential support to cope with both work and personal challenges; (3) feeling undervalued by the wider organisation, with a lack of investment including pay, facilities, and opportunities for development. These factors combined to create a situation of high stress and low job satisfaction, with many HCAs expressing an intention or desire to leave, particularly when the pay is similar to much less demanding jobs in other sectors.

**Conclusions:**

HCAs are a diverse group but many reported job dissatisfaction and feeling undervalued by the organisations they work for, with some struggling to cope with the cost-of-living crisis. Improved role boundaries, career pathways, and appreciation of the role such as reward and recognition schemes, could help retain this key staff group who provide a large proportion of essential patient care.

**Supplementary Information:**

The online version contains supplementary material available at 10.1186/s12913-025-12665-1.

## Background

In the UK, healthcare assistants (HCAs) provide direct patient care under the supervision of registered nurses and other clinicians. In contrast to the EU, HCAs in the UK are an unregulated workforce with no mandatory training or qualifications [1]. These staff are referred to as ‘unregistered’, with ‘registered’ staff being those registered with a professional regulator such as the Nursing and Midwifery Council or General Medical Council. Multiple job titles are used including support worker, recovery worker and nursing assistant; we use the term ‘HCA’ to encompass all non-registered clinical staff who provide direct patient care in a mental healthcare setting [2].

The HCA role varies considerably depending on the setting, particularly between inpatient and community settings, but can include observing and recording patients’ condition (e.g. through measuring vital signs and observing mental state) and other clinical duties; providing reassurance and ensuring safety; and encouraging and supporting independence and community engagement. A large proportion of HCAs’ time is spent in direct contact with patients and their relatives and carers. In 2013 a large review of HCAs in the UK [3] reported that they spent more time at the bedside than registered nurses and are therefore a key provider of clinical care.

In 2021, HCAs were estimated to make up around 31% of all clinically based National Health Service (NHS) staff. This proportion may now be even higher, as numbers of unregistered staff have increased more quickly compared to registered staff [4, 5]. However, HCA retention rates are lower than those of registered nurses, which is a matter of significant concern given the amount of essential care they provide [5]. According to NHS Digital figures for 2019, the average annual leaver rate for “support staff to doctors and nurses” was 13.4% [6]. In 2019, NHS England (who provide national leadership for NHS organisations) launched the “Healthcare Support Worker Programme”, one of whose main goals was to improve recruitment rate for HCAs, recognising that this group can move relatively easily to other sectors such as retail and hospitality, where pay in the UK is comparable or better [7]. Due to the vital role they play in delivering patient care, an improved HCA recruitment strategy was also considered to be crucial to the NHS’ COVID recovery strategy [7].

High turnover of HCAs impacts at multiple levels. At the macro level, the NHS is losing experienced staff, leading to increased costs for recruitment and training [8]. At a micro level, it negatively affects patient experience due to understaffing and lack of continuity of care. Loss of staff also increases pressure and workload for those remaining, which in turn makes them more likely to leave [9]. These impacts make HCA retention a significant priority.

The reasons for poor retention however are not well understood. Compared to sectors such as retail or hospitality, HCAs are poorly paid for what is often a very demanding role with limited career progression opportunities. HCAs and nurses in the UK report the lowest satisfaction with pay (13%) relative to other NHS staff [10, 11]. Staff surveys show that HCAs experience high levels of harassment and bullying and frequently report feeling undervalued [12], and higher levels of discrimination than registered nurses or doctors [13]. Additionally, HCAs are frequently more ethnically diverse than other staff groups (although like nursing), and staff surveys find higher levels of harassment and discrimination amongst BME staff in particular. It is not clear if these factors explain all the variation in retention or if other factors such as low position in the hierarchy are also significant [14]. The problem is not confined to the UK: in Ireland, nursing assistants in care homes listed low pay, workload, work-life balance, management support and job satisfaction as the main factors affecting their intention to leave [15]. An international review of HCAs working conditions found job satisfaction, supportive supervision and satisfaction with pay and additional benefits all reduced intention to leave [16].

Whilst these problems are significant across the healthcare sector, they are particularly acute within mental health, where overall vacancy levels are higher than elsewhere in the NHS [17]. Despite this, we have not identified any studies specifically exploring the factors affecting retention of HCAs in the mental health sector; this is therefore a key area for exploration.

## Methods

### Aim

This study aimed to investigate the experiences of HCAs working in mental health settings, specifically their perceptions of the factors they consider impact retention. It was undertaken as a further phase of a larger study exploring the factors affecting retention of registered staff including nurses, doctors, psychologists and allied health professionals (publication forthcoming). During this earlier phase we identified the need to explore the perspectives of unregistered staff, which led to this second phase being undertaken.

### Recruitment

We approached three mental health Trusts in England where we had recruited registered staff in the first phase of the study. These varied in location, retention rate, staff satisfaction, and quality inspection ratings (in the UK, the Care Quality Commission (CQC) undertakes and publishes online regular inspections and rating of health and social care services) (see Table 1).

**Table 1:** Characteristics of the included trusts

Location	1 Northern England 2 Southern England
Rural/urban footprint	1 Rural 2 Urban
CQC rating at the time of the research	1 Inadequate 1 Good 1 Outstanding
Leaver rate (all staff)	10.1% 12.0% 16.0%
Staff satisfaction rate	1 Very low 1 Neutral 1 Very high

Recruitment took place between December 2022 and May 2023. Each Trust’s Research and Development (R&D) team were responsible for emailing HCA staff groups either directly or via team leaders, with emails containing an introduction to the research and links to the participant information sheet and consent form. As many HCAs spend limited time on computers, we also provided posters to display in staff and clinical areas, which had a QR code linking to the study information. Trust R&D staff also attended staff meetings and research away days and circulated information via social media.

### Sampling

All non-registered staff who were patient-facing and based in adult or older adult inpatient or community mental health teams were eligible to participate. Those working in child or adolescent services were not eligible. As job titles varied, no job title was excluded provided they met the role description. Participants had to be employed by the NHS Trust rather than a third-party provider; for this reason, bank staff were included but not agency workers. Participants had to be in paid roles, volunteers were not eligible.

We aimed to interview 12–15 participants from each of the three Trusts, with a majority from inpatient settings where most HCAs are located. We aimed to achieve a representative sample of HCAs, with a particular focus on ethnic diversity in order to reflect the composition of this staff group [14]. To achieve this, we asked R&D teams to focus on recruiting via minority ethnic staff networks.

### Ethics

Institutional Research Ethics Committee (reference 037255) and Health Research Authority (Reference 21/HRA/0011) approvals were received prior to data collection. The NIHR Clinical Research Network portfolio supported this study. This research was carried out in accordance with the principles of the Helsinki Declaration.

### Data collection

Four members of the research team conducted semi-structured interviews, which were undertaken remotely using MS Teams, Google Meet or telephone, depending on the participant’s preference. The interview schedule (see Supplementary file) was based on a realist review of the factors affecting retention of UK mental health staff previously conducted by the team [18] and included questions exploring how a range of factors affected participants’ own intention to leave or stay, and retention of HCAs in general. These included workload, job satisfaction, teamwork, supervision, development opportunities, physical working environment, relationships with management and overall leadership, together with any additional issues highlighted by participants. We also explored perceived differences between HCAs and registered staff who had been interviewed in the previous stage of the study, particularly nurses. Interviews lasted between 45 and 60 min and were recorded and transcribed verbatim.

Research staff were completely independent of the organisation employing the HCAs and participants were told their responses would remain anonymous. Participants were asked to be completely honest. Although the possibility of power imbalance and the social desire to give the ‘right’ answer is hard to completely remove, participants did give positive and negative examples implying they felt comfortable to disclose this.

### Analysis

Three members of the team who had conducted the interviews undertook the analysis using framework analysis [19] and used Quirkos software for coding. The coding framework was iteratively developed from the realist review [18] and interviews with registered staff (publication forthcoming) in earlier phases of the project. The methods and processes used to generate this frameworkare described in full in our previous publication [18]. The framework was applied to the HCA data, which led to a very few minor modifications: removal of some unused codes, slight changes to the names of a small number of codes, and the addition of a code comparing experiences of registered and unregistered staff. Staff met regularly to review coding and discuss and resolve areas of uncertainty or disagreement.

## Results

An initial 49 people expressed an interest in the study, and a total of 31 participated in interviews. Of the 18 who were not interviewed, 12 did not respond to attempts to contact them, four withdrew, two were not eligible (one was a registered staff member, one worked for children’s services). Despite targeted efforts, we were only able to recruit four participants from minority ethnic backgrounds. The number of participants varied considerably between the three Trusts, with a very low response rate in one Trust. The demographic data and number of participants from each Trust is shown in Table 2.

**Table 2:** Interview participants’ demographic data

Category		Number (%)
Trust	A	4 (12.9)
	B	16 (51.6)
	C	11 (35.4)
Gender	Female	20 (64.5)
	Male	10 (32.2)
	Other	1 (3.2)
Ethnicity	White British	24 (77.4)
	White other	3 (9.7)
	Black, Asian and Minority Ethnic	4 (12.9)
Workplace setting	Inpatient	14 (45.2)
	Community	17 (54.8)
Qualification	None	8 (25.8)
	NVQ	11 (35.5)
	Diploma	3 (9.7)
	Degree or higher	9 (29.0)

Participants’ job titles included: support worker, recovery worker, care assistant and nursing assistant. There appeared to be no relationship between job title and pay band or participant qualifications. Most of the participants had a qualification (≈ 75%). Many had the National Vocational Qualifications (NVQs) either in healthcare or in specific areas related to their role such as dementia or addiction. Several participants were educated to degree or diploma level (*N* = 11, 36%).

**Fig. 1:**
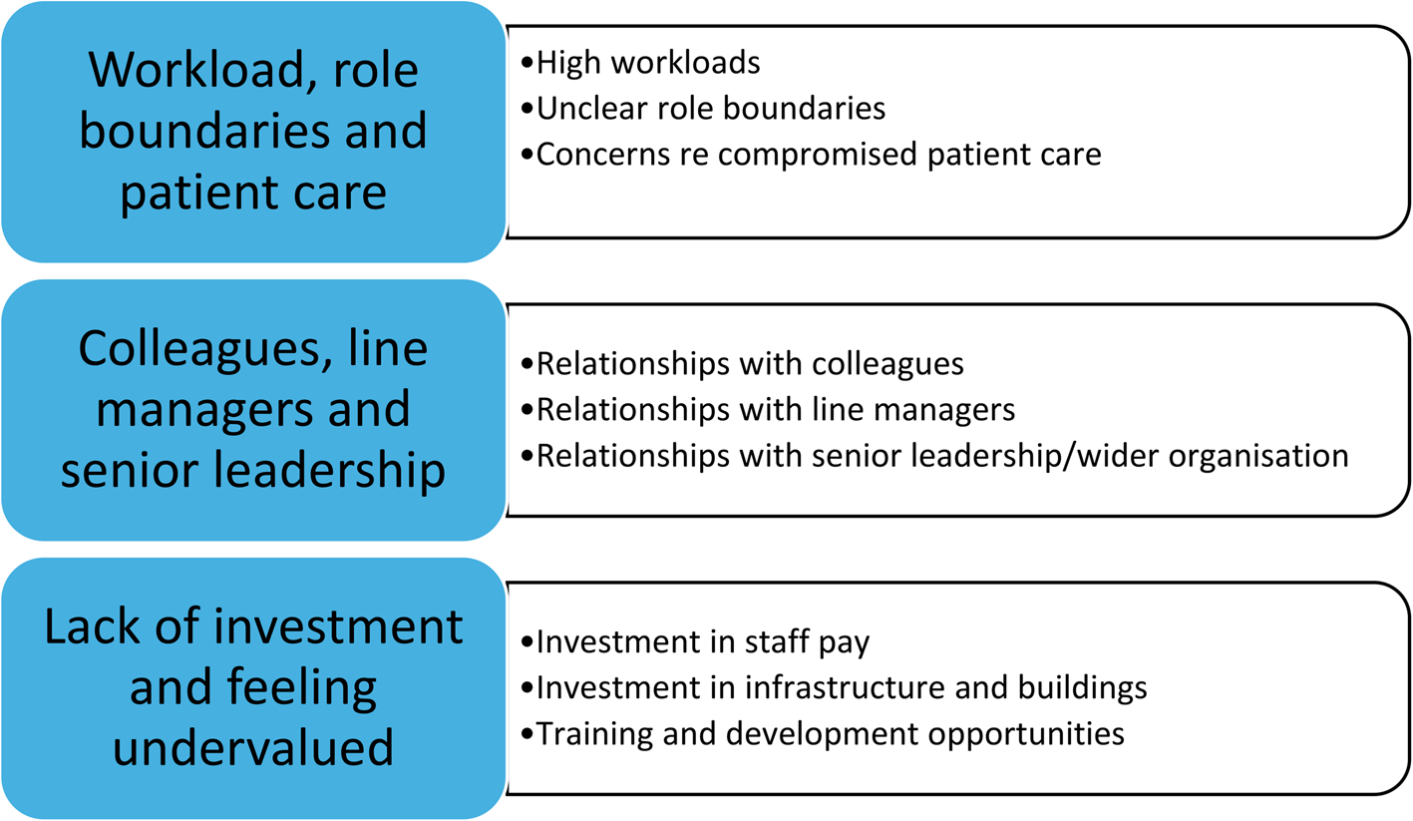
Themes and subthemes

HCAs highlighted a range of factors which impacted on their satisfaction and desire to stay in their role, which we grouped into three main themes: the challenge of high workload and unclear role boundaries, and the impact of this on patient care; the importance of relationships with managers, colleagues and senior leadership; and lack of investment in staff, including pay, buildings and infrastructure, and training and development opportunities (see Fig. 1 for Themes and Subthemes).

### High workload and unclear role boundaries, and the impact on quality of care

HCAs in all settings frequently identified high workload as a key issue, often a result of ongoing understaffing and high patient acuity, which impacted them in a variety of ways. In community settings, high overall caseload within teams led to delays in assessment and care to patients, with consequent concerns about risk and safety.

*we don’t have kind of like a cap on how many patients we can get referred …., so some days the expectation to see a certain amount of people with various different risks and various different needs in terms of paperwork etc can feel quite dangerous. C04*

In inpatient settings, understaffing led to HCAs having to undertake duties that were not what they had been employed or felt comfortable to do. They frequently felt unable to use their skills, which led to stress and frustration. A wider concern highlighted by many was being expected to work beyond their role boundaries, often with more responsibility, lack of appropriate training and no additional pay, and the stress, anxiety, and dissatisfaction this created.

*I had to jump into the numbers and I couldn’t do my life skills job. And it’s been like this on most days. A04*

*I think one of the things that feels quite*,* that’s quite a big stress I think is feeling that the boundaries of my role are kind of becoming blurry*,* in that I find myself taking on things that make me stop and think*,* hang on a minute*,* is that what I should be doing*,* is that even safe*,* am I actually being pulled into something that I shouldn’t be pulled into? C07*

*my colleagues*,* they do exactly what the band 6 does and they get paid on [band] 4 C12*

The excessive workload and pressure to work outside their role boundaries also contributed to concerns about the quality of care they, and the service as a whole, were able to offer. Whilst a few considered they were able to deliver good care, many felt that they were ‘letting patients down’ and that the quality of care was constantly jeopardised, with some reporting concerns for patient safety. The stress of working in an environment of high risk, and disillusionment at being unable to provide good care and do the job they wanted to, all impacted on HCAs’ motivation and job satisfaction, making some want to leave. Many described situations likely to cause moral injury.

*I don’t think we give bad care*,* but I don’t feel that we give the care that we could because it is so hard to you know*,* when you’re deciding who needs to be seen and you’re having to prioritise and rank people because there’s simply not enough staff B09*

Whilst HCAs identified many challenges shared by other staff groups, they also reflected on areas of difference in their experience, particularly compared to nurses, who they had the most contact with. Many observed how the additional accountability, responsibility, and administrative burden carried by nurses significantly added to the stress they were under, including the fear of losing their registration.

*it’s stressful for the nurses*,* the fact that they*,* if there’s a mistake*,* incident reports get written and things you know*,* they could lose their PIN [registration number] so trying to deliver a standard of care when you’re up against deadlines and time limits and maybe not enough staff on shift*,* that’s where you get the stress B15*

*I think there’s non-qualified*,* there’s nothing to answer to potentially so therefore you know*,* if you can go under the radar a bit*,* A02*

### The impact of relationships with colleagues, managers, and senior leadership/the wider organisation

Participants described the importance of relationships with their immediate colleagues and line manager, as well as the impact of their perceptions of senior leadership. In relation to line managers (e.g. team leader, ward manager), several key aspects were highlighted. Staff valued managers who offered support both directly related to their work and their wider wellbeing.

*So*,* she listens*,* she deals with you attentively*,* she doesn’t forget and she makes sure she does things that you ask sort of quickly*,* or will give you a response if she can’t. Checks how you are*,* always says hello. B08*

*I want to feel that someone else is listening to my decision making and not just assuming that I know what I’m doing all the time because I don’t feel like I do. C07*

*so my job satisfaction now being managed with a fantastic manager*,* having all the proper support at my level makes me feel like I’m of value of the Trust. A02*

Helping people maintain the boundaries of their role was seen as key, given the challenges highlighted in the previous theme. Being well-supported helped HCAs feel valued and motivated and, for some who were managing challenging personal circumstances, was key to being able to stay in their role.

In contrast, where this support was not in place, or managers were perceived as distant or lacking in competence, this could have a significant impact on retention.

*the reason people leave is because our manager is totally detached from what is happening on the floor C12*

*you’ve got a load of team leaders who’ve got no idea of how to manage staff because they’ve done no management qualification to get there*,* in this service anyway. B09*

HCAs also emphasised the importance of supervision, but how frequently this was deprioritised due to a lack of time and staff shortages.

*I’m entitled to a supervision but they’re so busy that I can’t*,* I daren’t be asking them and I’m usually busy as well B20*

Good relationships with other immediate colleagues were also frequently highlighted and could in some instances mitigate a poor or absent manager. Supportive relationships helped people cope with the challenges of the work, create a sense of shared values and purpose, and made them feel valued and respected. Where these were absent, or in some instances negative, this could significantly impact wellbeing and retention.

*I think my team actually is one of the main reasons for retention at the moment actually*,* because I have been thinking about leaving and like part of it is that I really enjoy the community that I work with*,* for the most part B06*

*I can feel like there’s a lot of gossiping*,* and that makes me*,* it makes me feel like I’m in school*,* and I don’t want it. It makes me want to leave. C09*

Regardless of the quality of relationships with immediate colleagues and managers, HCAs also spoke about their lack of confidence in or connection to senior leadership. Most described feeling that senior staff lacked understanding of the day-to-day work with patients, and did not listen to or care about their experiences and challenges.

*I think that people in senior positions in my Trust are quite out of touch you know*,* we don’t have a lot of contact from people in senior positions*,* especially as support workers…you don’t get included in anything at all….B11*

### Lack of investment in staff including pay, infrastructure, and opportunities for development

Many HCAs described ways in which they felt the organisation did not value or invest in them, and the impact of this on their job satisfaction and intention to stay. One key area was low pay, with some reflecting how they could get equivalent or better pay in retail or hospitality settings but with far less stress and challenge. A minority described poverty, including the need to work multiple jobs, use foodbanks and even reported colleague homelessness.

*you’re dealing with people who are trying to commit suicide*,* ligatures and all sorts*,* on the job*,* the alarm’s always going off and these are people are getting paid absolute crap [sic] C03*

*like today I’ve fed my children*,* but I can’t afford to eat myself so*,* you know I am weighing it up to myself like why am I doing this job because I work so hard with some of the most vulnerable people in society? A06*

The physical environment and resources in the workplace could also contribute significantly to people’s motivation and sense of being valued. Many described poorly maintained buildings which were not fit for purpose, including poorly equipped kitchens and limited office space. In addition to the discomfort and low morale this caused, this raised wider concerns about staff and patient safety.

*Oh my god the physical environment is awful*,* it’s old*,* it’s not maintained. […] So that makes a massive difference because you’re coming into a dirty old building where it’s cluttered and it’s chaotic and then you’ve also got this stress of dealing with patients so it can be really hard to concentrate and focus. C01*

*We don’t have enough space*,* we don’t have enough decent equipment and I have felt*,* I’ve just had times where I’ve felt a bit forgotten because I’ve just been dumped in a corner. B14*

A final area of concern related to opportunities for training and development, with differing views amongst HCAs reflecting their own plans and motivations. Some participants were not seeking career progression, often because of other personal demands such as family caring responsibilities or their own mental health.

*A lot of us in lower bands are carers. B14*

*it’s my comfort blanket. I know the job*,* I know the staff*,* I have suffered with my mental health in the past and people know me and I know the people. B21*

In contrast others, particularly those with higher levels of educational qualification, considered the lack of opportunities for development a significant factor in their motivation to move on from the role. Many felt frustrated by the lack of training, with some stating that this meant the role was only a ‘stepping stone’ in their career trajectory.

*they need to really think about how they can make the other non-nursing staff feel valued and that’s opening up opportunities that people can progress through because you’re hard strapped to progress anywhere without being a nurse. B9*

*the only reason that I’m staying is to get my hours […] because I’m still trying to get those hours*,* so that if I do decide to go off and do something*,* I have them under my belt. C09*

Others considered the potential for development existed but expressed reservations about progressing to a higher band having observed the pressure their colleagues faced.

*We come into these roles a lot of us wanting to work up*,* but when you start to see looking up at your colleagues that all of them are really unhappy*,* then you just think well I don’t want to do that anymore. C01*

A further factor highlighted by some as contributing to the sense of not being valued by the organisation was the categorisation of HCAs as ‘unqualified’ (i.e. not a qualified nurse), despite many having higher qualifications or professional training. They felt at the bottom of the professional hierarchy and not seen as legitimate.

HCAs also reflected on differences in opportunities for development and change between themselves and registered nurses. Some considered that non-registered staff had more choices available to them outside the organisation, partly due to the low pay. They reflected that registered staff may also be more reluctant to leave due to the time and effort invested in their training. In contrast, others considered a greater range of options in healthcare were available to registered nurses, including opportunities within the private or voluntary sector, or taking agency work.

## Discussion

Whilst a few HCAs expressed satisfaction with their work experience, the majority highlighted a range of issues that significantly reduced their job satisfaction, wellbeing, and intention to stay in the role. High workload and unclear work boundaries led to significant levels of stress, including concerns for patient care and safety. Supportive relationships with line managers and colleagues were frequently a vital resource to help them manage these challenges, sometimes making a difference to their decision to stay or, where these were absent, contributing to their desire to leave. Irrespective of these immediate relationships, many described feeling undervalued by senior leadership and spoke of the demoralising effects of lack of organisational investment, including low pay, poor infrastructure, and lack of opportunities for development and progression, organisational factors also noted by registered mental health nurses as affecting their intention to leave [20]. These factors combined to create a situation where HCAs were frequently taking on additional responsibility and duties with limited training and support, whilst working in unsatisfactory environments for similar pay to supermarket staff. As a result, many described feeling undervalued, with a lack of recognition for the increasingly demanding work they undertake. This mirrors other research investigating rising levels of burnout and poor wellbeing among mental health staff [21].

Many participants described considering or having a firm intention to leave, but there was variation within the group which appeared to relate to individuals’ particular circumstances. Those with higher levels of qualifications often saw the role as a step in their career trajectory, and the lack of training and progression opportunities contributed significantly to their desire to leave, mirroring findings in an Italian study [22]. In contrast, others described personal challenges that meant they were unwilling to move out of the familiar role, particularly where they had supportive colleagues and managers. This highlights the diversity of motivations within this group, and the need for organisational flexibility in addressing them to improve retention. This is particularly important given the projected increased demand for HCAs alongside high rates of turnover, which could exacerbate existing HCA shortages [23].

It was notable that HCAs highlighted their own personal challenges more frequently than registered staff interviewed for the previous phase of the study (publication forthcoming). This may be due to this group experiencing a high rate of issues and potentially staying in lower banded roles as a result, or greater acceptability of acknowledging personal difficulties, something which is not encouraged within the nursing or medical culture. Additionally, issues related to low pay and even poverty were reported more frequently amongst HCAs. Given their lower banding, this is perhaps unsurprising, but the situation is likely to have been exacerbated by the cost-of-living crisis that has occurred since the previous interviews were undertaken. However, similar concerns about pay were reported in 2010 [24].

Similarities with previous work undertaken to understand retention within the registered adult nursing workforce are clear. Cowden and Cummings [25] highlight a range of organisational, environmental, and behavioural factors that contribute to intention to stay for this group. Many of these resonate with features identified by HCAs, including leadership, praise and recognition, supervisor support, staffing levels and work group cohesion. The notion of supportive leadership and close colleagues is also similarly identified within cohorts of registered mental health nurses: Holmberg et al. [26], highlight the importance of satisfying relationships as providing a *‘safe haven’* (p586) and an important factor in improving retention.

When participants made comparisons with registered nurses, these related to scope of practice and role boundary blurring. Our findings suggest that HCAs are very likely to work beyond their role descriptions, with actual work not matching job descriptions. As a result, their contribution is often undervalued. A lack of national standards for HCA role design has led to the intensification of work [27]. A mapping exercise of the HCA role in the EU found that HCAs are more likely to be defined *“in terms of knowledge and skills*,* often at a basic instead of more specialized level*,* and much less so in terms of competences”* ([20], p1109). This contributes significantly to undervaluing the HCA role, which then impacts on motivation and consequent retention.

Many HCAs also described how high workloads meant they were unable to provide the patient care they wanted to, and which had originally motivated them to come into the role. This led to dissatisfaction, but also raised concerns in some instances about patient safety. Other studies have also found that it is not staff shortages and patient demand per se that cause job dissatisfaction, but the consequences of this [12]. When the conditions are inadequate to provide safe patient care, staff experience significant stress and anxiety, alongside the demoralising impact of feeling they are letting patients down. Long term exposure to this can lead to moral injury [28–31].

In this challenging context, participants emphasised the importance of relationships with colleagues, particularly managers, in mitigating the impacts of high workloads and short staffing and therefore improving retention. Other studies have also found that improving teamwork positively impacts on resilience [32]. Whilst some HCAs reported and valued helpful and supportive managers, others described distant, ‘hands off’ approaches which lacked understanding of their situation, or managers who lacked the skills and knowledge to provide appropriate support. The significance of this issue has been raised recently in the UK government NHS workforce plan, where organisations are instructed to take a more active role to promote and invest in the wellbeing of their staff, to improve retention and recruitment [33]. Previous research has found leadership training can be transformative if there is an enabling environment for managers to put their training into action [34].

Finally, some acknowledgment of the geography of the organisation is important. Different challenges exist for rural and urban employers. Many rural organisations struggle to recruit as the locale may not be seen as enticing to younger staff, but many urban organisations struggle to retain as there are many alternatives nearby. This is an emerging area in the UK [35] but has been considered in other countries [36].

### Limitations

We experienced significant challenges with recruitment, particularly in one Trust and, despite focused efforts, we particularly struggled to recruit staff from minority ethnic backgrounds. Although we extended the recruitment period by eight weeks, none of the Trusts recruited to target. Several factors may have contributed to this. We were aware that many HCAs do not use email in their day-to-day work, and that generic email is frequently deleted without being read; as this was our primary recruitment tool, many were probably unaware of the study. We were unable to spend time on site which may have enabled us to build rapport and confidence with potential participants. This may be particularly important with this group of staff, who often do not perceive their views to be valued and may have been reluctant to speak out. Additionally, HCAs contain a disproportionately large number of people from minority ethnic groups, a population that frequently are not well represented in research for a complex range of reasons, including lack of trust and confidence in institutional processes due to the discrimination experienced. As our recruitment methods relied on communication from those institutions i.e. the Trusts, this may well have been a significant factor that negatively impacted people’s willingness to engage in the research. Future projects should budget for additional time and resource to build personal connections with communities of staff and encourage snowball sampling. Local knowledge about informal networks, rather than Trust networks, may be helpful as they may be seen as more trustworthy than those working for the organisation.

A possible additional factor affecting recruitment was that we did not offer any form of financial incentive to participate; this may have been particularly significant for this low-income group of staff. These considerations are supported by our previous experience of recruiting registered staff from the same trusts, where we successfully recruited but had a similar unrepresentatively low proportion of minority ethnic staff.

As with the previous phase of the study, we were unable to interview HCAs who had actually left, as we had no means of accessing them. It may be that some people remain in post despite their dissatisfaction, or ultimately find it difficult to find other suitable work, but as we were only able to conduct interviews at a single point in time, we are unable to explore these questions further. However, a number of participants reported actively seeking other work or described job changes that ex-colleagues had made to move out of mental healthcare.

### Recommendations/implications

Retention strategies for HCAs should focus on better definition, recognition, and improved appreciation of the role and its pivotal contribution to mental health services. This appreciation should be reflected accordingly through investment in staff, including adequate training provision, improved leadership training for managers and leaders, supervision, reward and recognition programmes, progression opportunities, and a living wage. Better workforce policies would benefit HCAs and the organisations who struggle to meet demand and retain staff. Where HCAs cannot be retained within this role, organisations need to consider career pathways which enable people to be retained within the organisation.

Further research is needed to inform and better define HCAs status, job roles, competencies, and qualifications. There is also a need to further explore the differing motivations with the HCA staff group and identify a range of strategies to better improve their retention. This needs to include recruitment of a more diverse and representative range of staff.

## Conclusions

Healthcare support workers are a diverse group, but many shared significant levels of job dissatisfaction caused by high workload and demands, feeling undervalued by the organisation, and in some instances lack of support from managers and colleagues. Some were struggling financially, exacerbated by the cost-of-living crisis, and considering moving to less demanding work with similar remuneration. Improved role boundaries, career pathways, and appreciation of the role could help in retaining experienced staff who provide a large proportion of essential care for patients.

## Supplementary Information

Supplementary Material 1.

## Data Availability

Anonymised data will be available on request. Request will require a protocol, research ethics committee approval and will be pending a data sharing agreement.

## Abbreviations

CQC: Care Quality Commission
HCA: Health care assistant
NHS: National Health Service
NVQ: National Vocational Qualifications
R&D: Research and Development team
