# Supplementary material for: Factors affecting the retention of healthcare assistants in English mental health services: a qualitative interview study
Source: BMC Health Serv Res. 2025 Apr 5;25:505. doi: 10.1186/s12913-025-12665-1 (PMC11972535; doi:10.1186/s12913-025-12665-1)
Supplement: Supplementary file 1 — Supplementary Material 1. [file 12913_2025_12665_MOESM1_ESM.pdf]

ROMHS – WP3 Case Studies: Pilot interview schedule V3

Introduction

- Any questions?
- Clarify re consent
- Confirm re recording
- Confirm re confidentiality – discuss recording
- Recap on purpose of project: to understand what factors affect retention of MH staff. Comparing different Trusts with different rates to see what makes the difference, and how we can learn from this to improve situation for everyone, develop guidance at national level.
- Explain format of interview: very open, some particular areas to explore but also interested in individuals' own perspective. We'll make sure we cover all the areas we need to, so just feel free to talk. Can either talk about own experience, or generically – up to them.

Questions

- Role in the Trust, how long been in this role? Any previous roles in Trust? How long in Trust overall?
- Demographics – gender, ethnic group or background (self-describe for both questions)
- Do you have any healthcare specific qualifications?
- Tell me about your role and how you came to it?
- Tell us a bit about who is in your team
- Do you have a standard contract or bank or both
- Any experience of working in any other Trusts/private sector? (just to see if this is something to pursue later, not discuss at this point)

- **What do you think works well/is a problem for the retention of clinical staff in your Trust? Or, in more simple terms – what are the things that make people stay or go in your Trust?**
  [See what comes from this question – let people identify their own priority areas rather than steer towards PTs at this point.
  Ensure conversation remains focused around non- registered clinical staff.]

- **We've identified six areas that seem to be important in relation to retention of staff. Some of these you've already talked about, but can you give me your thoughts on the others, and how much you think they affect retention? These are:**
  - Workload, staffing levels and staffing mix
    [relate to nursing staffing levels, appropriateness of responsibility levels]
  - Staff perceptions of the quality of care they are able to deliver
    [Whether you feel you're able to do a good job personally but also whether

you feel the Trust/unit you're in is doing so.
Explore something around relationships with patients, impact on these and how this affects staff experience.]

- Team relationships and cohesion
  [Includes, involvement in decision-making, being listened to.]
- Leadership and organisational culture
  [This is about 'top level' management rather than ward/team manager, though they may have things to say about this local level as well.]
- Development/training opportunities
- Supervision

- How do you think these factors relate to job satisfaction?
- And how do you think job satisfaction relates to intention to leave?

- What is your built environment like?
- How much do you think the local context affects whether people stay or not?
  [Things to consider: local labour market – other options, demographics e.g. population age/mobility, immigration/visa status etc.
  Also physical environment – local area, buildings, resources.]
- How does your experience in this Trust compare to any other places you've worked? What's better or worse? Comparisons with other people you know in other Trusts?
- Variation across the Trust? Different services etc? If so, why?
- What impact do you think COVID has had/will have on staff retention, if any?

- What keeps you in post/makes you stay? Are you thinking about leaving? What would change this/make you feel you wanted to stay? (including registered training)

- Do you think there are any differences between HCA/SWs and registered staff when it comes to retention?

- We have found it difficult to recruit HCAs, particularly from the wards. Do you have any thoughts on why that might be and what we can do differently?

Completion

- Thanks
- Explain what will happen next
- Offer brief summary of findings
- Reiterate to the participants not to download recording, will be deleted from Teams once downloaded by interviewer.
- Any questions?
